# Sustainable Developmental Goals interrupted: Overcoming challenges to global child and adolescent health

**DOI:** 10.1371/journal.pmed.1003802

**Published:** 2021-09-28

**Authors:** Zulfiqar A. Bhutta, Kathryn M. Yount, Quique Bassat, Caitlin E. Moyer

**Affiliations:** 1 Centre for Global Child Health, the Hospital for Sick Children, Toronto, Canada; 2 Institute for Global Health & Development, The Aga Khan University, Karachi, Pakistan; 3 Hubert Department of Global Health, Emory University, Atlanta, Georgia, United States of America; 4 ISGlobal, Hospital Clínic—Universitat de Barcelona, Barcelona, Spain; 5 Centro de Investigação em Saúde de Manhiça (CISM), Maputo, Mozambique; 6 ICREA, Pg. Lluís Companys 23, Barcelona, Spain; 7 Public Library of Science, San Francisco, California, United States of America and Cambridge, United Kingdom

Few predicted, in September 2015, when the millennium development goals (MDGs) closed and the Sustainable Development Goals (SDGs) were signed into a global compact, that we might soon face the largest global public health challenge in over a century. Not only has COVID-19 killed nearly five million people to date [1], and upended global economies, but it has also seriously impacted child health and development across the world, especially in low- and lower middle-income countries (LLMICs) [2]. For the first time in living memory, over 1.6 billion children are out of school [3] and efforts on reopening schools have sparked rancorous and divisive debates. There are legitimate concerns that COVID-19 has negatively impacted progress in achieving the SDGs globally, and that urgent redirective strategies are needed before hard-earned gains from the 2000–2015 MDG period are reversed [4].

In October 2020, we announced a Special Issue of *PLOS Medicine* devoted to themes of child and adolescent health in the context of the SDGs [5]. At the time of our call for papers, the world was consumed by efforts to counter the COVID-19 pandemic, and has remained so; however, we set out to solicit papers on topics and problems that were left unaddressed at the end of the MDG period in 2015, issues that preceded the current pandemic and that are likely to persist after the current crisis abates. Our objectives were to encourage submissions pertaining to specific areas related to child development, adolescent health, and the social determinants of health. The depth and breadth of submitted articles, now reflected in the accompanying research papers, remarkably demonstrates the global significance of these issues and the multi-faceted approaches used to address them. The Special Issue represents a kaleidoscope of papers that span a range of topics and areas including child health and its drivers, including newborn health, and immunizations and other strategies to combat treatable and preventable infections; nutrition support and innovative interventions to improve infant and child survival and growth; adolescent health and outcomes, in areas such as gender equity, HIV, injuries, peer/school-based violence, and mental health; and the socio-contextual determinants of health relevant for all stages of childhood and adolescent development, including, for example, those related to conflict, environmental conditions, and the family and parenting environment.

This Special Issue has contextual relevance in light of the broad spectrum of direct and collateral effects wrought by the COVID-19 pandemic on social, economic, and health-related conditions [6]. These effects, direct and indirect, can be grouped broadly in relation to current controversies, such as the upsurge in direct infections among children and adolescents in the wake of the global spread of the B1.617.2 (Delta) variant [7], and the uncertain relationship of community spread with school closures and reopening [8]. Currently, the global inequity in COVID-19 vaccine availability and uptake as well as the debate on deferring vaccinations for children and adolescents in high-income countries (HICs) to vaccinate adults in LMICs, also pose major ethical dilemmas [9]. In the medium term, building back better and fairer maternal and child health services [10] will be critical to regain the momentum needed to reach the SDG targets. In the longer term, sustained investments across multiple sectors and the continuum of age across childhood and adolescence are needed to support and sustain universal attainment of physical, academic, and social milestones. The full extent of short-, medium- and long-term repercussions of the COVID-19 pandemic on child and adolescent health remain to be seen. It will be crucial to assess emerging interactions between ongoing child and adolescent health issues and COVID-19-related effects.

Post-COVID-19, there is the potential for worsening of many of the pre-existing challenges, which will be crucial to address in the rebuilding process. The research articles highlighted in this Special Issue serve to illustrate current challenges, and innovative strategies to target and improve child and adolescent health. We also discuss examples of research relevant for these challenging contexts.

## Poverty

Poverty can have a profound impact on child health outcomes, and according to an analysis by Save the Children and UNICEF, the percentages of children without access to education or health services globally are estimated to have increased to 56% as a result of the COVID-19 pandemic [11]. Integrated cash-transfer programs exist in many countries to lend economic and social support and to lessen the negative consequences of poverty and limited social protections on child and adolescent health outcomes. Conditional cash transfer (CCT) programs can have a positive impact on child and adolescent health outcomes by the direct provision of financial support and the condition that beneficiary families satisfy health requirements, such as obtaining routine childhood vaccinations. Integrated cash-transfer programs also may include elements to improve aspects of child and adolescent development directly, via interventions targeting health, education, or nutritional outcomes. In their accompanying paper, Madison Little and colleagues conducted a systematic review and meta-analysis to assess the existing evidence supporting whether these “plus” components together with cash transfers provide additional effectiveness in terms of achieving child health and wellbeing goals in line with the SDGs [12]. The findings suggest that the combination of cash with food transfers have additional benefits for child growth when compared to cash transfer alone. The results were also suggestive of benefits for cash and food transfer in crisis contexts, and cash transfer combined with primary healthcare intervention. However, the authors point out a relative paucity of studies comparing these cash-plus programs to cash transfer alone, indicating that more research is needed to evaluate fully the benefits of these interventions. Further investigation of the effectiveness of cash transfer programs in additional contexts may be useful to mitigate the negative impacts of the current pandemic, and to inform the rebuilding process.

## Pollution and climate change

Two new studies offer insights into the relationships between exposure to poor air quality and extreme weather conditions, both expanding concerns across the globe, and adverse perinatal and childhood health outcomes. In a systematic review and meta regression, Rakesh Ghosh and co-workers [13] revealed that ambient and household PM_2.5_ levels are associated with a higher risk of children born with low birth weight and born preterm across multiple countries. The analyses also suggested that a large proportion of infants with low birth weight and preterm births globally may be attributable to PM_2.5_ levels. These findings shed light on the potential impact of worsening air quality on perinatal and child health. While some of the repercussions of a changing climate on child health are easy to foresee, it is also clear that environmental changes can have less intuitive but potentially devastating consequences for health outcomes. Jason M. Nagata and colleagues investigated the associations between drought and child vaccination coverage in 22 countries in sub-Saharan Africa, using Demographic and Health Survey data from 2011 to 2019 [14]. As infections contribute to the global burden of child mortality worldwide, vaccination is crucial for protection from a wide range of childhood illnesses, and the findings of this study suggest that drought conditions may hinder vaccination coverage, an important consideration as weather patterns are disrupted and become more extreme, globally. Together these studies underscore the implications of a changing climate for child health and emphasize the importance of adopting measures to combat deterioration of the environment.

## Violence and conflict

Despite improved survival in recent decades, an unfinished agenda remains of residual maternal, newborn and child mortality, now increasingly dependent upon implementation in difficult contexts, such as impoverished urban neighborhoods, conflict zones, and pandemic conditions. Maternal and child mortality rates are higher among those living in countries with conflict [15]. A report by Anu Rammohan and co-workers revealed associations between exposure to violent conflicts and take-up of maternal health care services, including antenatal and postnatal care, across countries [16]. Among nearly half a million women from 49 countries between 1997–2018, the study found that about 40% were exposed to violent conflict during pregnancy, and the odds of utilization of maternal continuum of care components were lower among those who reported being exposed to any violent conflict. This finding speaks to how exposure to violent conflict has the potential to impede progress in achieving maternal and newborn care goals, and targeted interventions in such settings may be necessary to improve access to antenatal and postnatal care.

## Psychosocial, academic, and gender-based challenges

Well before COVID-19, poor mental health was fast emerging as a major non-communicable disease of relevance to children and adolescents, with far-reaching and long-term consequences, including those related to bullying and exposure to other forms of violence. Exacerbating that trajectory is the differentiated context of the pandemic [17], and, therefore, a better understanding of contributors to mental and socioemotional health issues is imperative at the present time. Several studies in this Special Issue illuminate the current challenges in this area, as well as inform future strategies to combat psychosocial and mental health issues going forward. Italo Lopez Garcia and colleagues conducted an economic evaluation of an effective parenting intervention to promote cognitive, receptive language, and socioemotional development among children under three years of age in Kenya [18]. The results suggested that a group-based intervention delivered by community-health-care workers was highly cost-effective, and the authors estimated a large potential return on investment when considering the potential impact of the intervention on lifetime wages and schooling costs. This is important, as it demonstrates the financial feasibility of implementing an intervention likely to be successful in enabling children to reach academic milestones.

Health and mental-health issues stemming from gender inequality are an area of special concern for adolescent girls globally. In a cross-sectional study conducted among adolescent girls enrolled in schools in western Kenya, Garazi Zulaika and colleagues found a number of contextual risk factors, such as experiencing harassment and sexual violence, associated with health and gender equality outcomes including sexual activity, HIV/HSV infection and adolescent pregnancy [19]. By describing the factors associated with these outcomes, it is possible to identify strategies to counteract contributors to adolescent mortality, including HIV and maternal factors among adolescent girls, and ultimately thereby, to improve health outcomes.

Now more than ever, it is critical to refocus the SDGs with children and adolescents at the center. To accomplish this refocus, it is necessary to recognize and attend to the health inequities among children and adolescents arising from differentiated exposures to dynamic social contexts around the globe, including urban poverty, environmental degradation and extreme climates, violence and conflict, and unsafe school, family, and social environments. There is a continued need for evidence-informed advocacy for action, and the studies we describe above, together with the rest of the informative research featured in this *PLOS Medicine* Special Issue, serve as examples of the evidence to support the need for driving child and adolescent health to the forefront. When we emerge from the COVID-19 pandemic, prioritizing these goals will be necessary while striving to rebuild a better, fairer, and more sustainable context in which children and adolescents can thrive. It is our hope that the research published here will inspire future work to understand the challenges facing the achievement of child and adolescent health goals, and to develop new and innovative strategies to overcome these challenges.

## References

[1] Johns Hopkins University. COVID-19 dashboard. Accessed Sept 3, 2021. https://coronavirus.jhu.edu/map.html

[2] Roberton T, Carter ED, Chou VB, Stegmuller AR, Jackson BD, Tam Y, Sawadogo-Lewis T, Walker N. Early estimates of the indirect effects of the COVID-19 pandemic on maternal and child mortality in low-income and middle-income countries: a modelling study. Lancet Glob Health. 2020 Jul;8(7):e901–e908. doi: 10.1016/S2214-109X(20)30229-1 Epub 2020 May 12. ; PMCID: PMC7217645 32405459 PMC7217645

[3] UNESCO. One year into COVID: Prioritizing education recovery to avoid a generational catastrophe. March 23, 2021. https://en.unesco.org/news/one-year-covid-prioritizing-education-recovery-avoid-generational-catastrophe

[4] United Nations. Sustainable Development Goals Report 2021. The Decade of Action for Sustainable Development Goals. https://s3.amazonaws.com/sustainabledevelopment.report/2021/2021-sustainable-development-report.pdf

[5] Bhutta ZA, Yount KM, Bassat Q, Arikainen AA. Revisiting child and adolescent health in the context of the Sustainable Development Goals. PLoS Med. 2020;17(10): e1003449. doi: 10.1371/journal.pmed.1003449 33125368 PMC7598466

[6] UNICEF. Direct and indirect effects of the COVID-19 pandemic and response in South Asia. March 2021. https://www.unicef.org/rosa/media/13066/file/Main%20Report.pdf

[7] American Academy of Pediatrics. Children and COVID-19: State level data report. https://www.aap.org/en/pages/2019-novel-coronavirus-covid-19-infections/children-and-covid-19-state-level-data-report/. Aug 28, 2021

[8] Irfan O, Li J, Tang K, Wang Z, Bhutta ZA. Risk of infection and transmission of SARS-CoV-2 among children and adolescents in households, communities and educational settings: A systematic review and meta-analysis. J Glob Health. 2021 Jul 17;11:05013. doi: 10.7189/jogh.11.05013; PMCID: PMC8285769. 34326997 PMC8285769

[9] Ledford H. Should children get COVID vaccines? What the science says? Nature. July 20, 2021. https://www.nature.com/articles/d41586-021-01898-9 doi: 10.1038/d41586-021-01898-9 34285402

[10] WHO. Building back fairer. http://www.emro.who.int/media/news/report-of-the-commission-on-social-determinants-of-health-in-the-eastern-mediterranean-region.html

[11] Save the Children & UNICEF. Technical Note: Impact of COVID-19 on multidimensional child poverty. September 2020. https://data.unicef.org/resources/impact-of-covid-19-on-multidimensional-child-poverty/

[12] Little MT, Roelen K, Lange BCL, Steinert JI, Yakubovich AR, Cluver L, et al. Effectiveness of cash-plus programmes on early childhood outcomes compared to cash transfers alone: A systematic review and meta-analysis in low- and middle-income countries. PLoS Med. 2021;18(9): e1003698. 10.1371/journal.pmed.1003698 PMC8478252 34582447

[13] Ghosh R, Causey K, Burkart K, Wozniak S, Cohen A, Brauer M. Ambient and household PM2.5 pollution and adverse perinatal outcomes: A meta-regression and analysis of attributable global burden for 204 countries and territories. PLoS Med. 2021;18(9): e1003718. 10.1371/journal.pmed.1003718 PMC8478226 34582444

[14] Nagata JM, Epstein A, Ganson KT, Benmarhnia T, Weiser SD. Drought and child vaccination coverage in 22 countries in sub-Saharan Africa: A retrospective analysis of national survey data from 2011 to 2019. PLoS Med. 2021;18(9): e1003678. 10.1371/journal.pmed.1003678 PMC8478213 34582463

[15] Bendavid E, Boerma T, Akseer N, Langer A, Malembaka EB, Okiro EA, Wise PH, Heft-Neal S, Black RE, Bhutta ZA; BRANCH Consortium Steering Committee. The effects of armed conflict on the health of women and children. Lancet. 2021 Feb 6;397(10273):522–532. doi: 10.1016/S0140-6736(21)00131-8 Epub 2021 Jan 24. . 33503456 PMC7612212

[16] Goli S, Rammohan A, Mavisakalyan A, Vu L. Exposure to conflicts and the continuum of maternal healthcare: Analyses of pooled cross-sectional data for 452,192 women across 49 countries and 82 surveys. PLoS Med. 2021;18(9): e1003690. 10.1371/journal.pmed.1003690 PMC8478181 34582443

[17] Chadi N, Spinoso-Di Piano C, Osmanlliu E, Gravel J, Drouin O. Mental Health-Related Emergency Department Visits in Adolescents Before and During the COVID-19 Pandemic: A Multicentric Retrospective Study. J Adolesc Health. 2021 Aug 5:S1054-139X(21)00393-1. doi: 10.1016/j.jadohealth.2021.07.036 Epub ahead of print. . 34462192 PMC8421028

[18] Luoto JE, Lopez Garcia I, Saya UY. Cost-effectiveness and economic returns of group-based parenting interventions to promote early childhood development: Results from a randomized controlled trial in rural Kenya. PLoS Med. 2021;18(9): e1003746. 10.1371/journal.pmed.1003746 PMC8478245 34582449

[19] Zulaika G, Nyothach E, van Eijk AM, Obor D, Mason L, Wang D, et al. Factors associated with the prevalence of HIV, HSV-2, pregnancy, and reported sexual activity among adolescent girls in rural western Kenya: A cross-sectional analysis of baseline data in a cluster randomised controlled trial. PLoS Med. 2021;18(9): e1003756. 10.1371/journal.pmed.1003756 PMC8478198 34582445

